# Supplementary material for: Brain-wide mapping of neural activity controlling zebrafish exploratory locomotion
Source: eLife. 2016 Mar 22;5:e12741. doi: 10.7554/eLife.12741 (PMC4841782; doi:10.7554/eLife.12741)
Supplement: Supplementary file 1. — DOI: http://dx.doi.org/10.7554/eLife.12741.029 [file elife-12741-supp1.zip › spontaneous-turning.html]

spontaneous-turning


# Intro¶

This notebook demonstrates an example voxel-wise regression analysis of turning-related neural responses in whole-brain lightsheet-imaging in zebrafish, to identify the anterior rhombencephalic turning region (ARTR).

The data for this example is available at **s3://neuro.datasets/ahrens.lab/spontaneous.turning/2/**, with the imaging voxel time series in a custom flat binary format well suited to parallel IO, and behavioral parameters in JSON. Data can be accessed and analyzed in Python as shown in this notebook.

This notebook was run using Spark 1.5.1 and Thunder 0.6.0 and other helper modules. This notebook assumes a Spark Context has been created and is available in the variable `sc`, see the Spark documentation for more info.

# Set up¶

In [1]:

```
from thunder import ThunderContext
tsc = ThunderContext(sc)
```

In [3]:

```
%matplotlib inline
```

In [4]:

```
import matplotlib.pyplot as plt
from showit import image
```

# Load the data¶

Specify the path (either a directory on a networked-file system, or a location on Amazon S3)

In [5]:

```
path = 's3n://neuro.datasets/ahrens.lab/spontaneous.turning/2/'
```

Load the data (the loading itself won't actually happen until later, when we query the data)

In [6]:

```
data = tsc.loadSeries(path + 'series')
```

Inpsect the data set to see some basic properties (some won't be available because we haven't computed them yet)

In [7]:

```
data
```

Out[7]:

```
Series
nrecords: None (inspect to compute)
dtype: float32
shape: None (inspect to compute)
dims: None (inspect to compute)
index: [   0    1    2 ..., 6562 6563 6564]
```

Load and inspect the parameters

In [8]:

```
params = tsc.loadParams(path + 'params/covariates.json')
params
```

Out[8]:

```
Params
names: ['turns', 'radius', 'theta']
```

Grab the parameters related to turning into a local array. This is a matrix of regressors, computed by downsampling, binning, and convolving raw electrophysiological signal (steps not shown here).

In [9]:

```
X = params['turns']
```

# Regression¶

First we import the appropriate class from Thunder

In [10]:

```
from thunder import LinearRegression
```

Create a model for linear regression

In [11]:

```
model = LinearRegression()
```

Do some basic temporal filter on the data set, including nonlinear detrending and converting into dF/F via normalizing. We compute the dimensions as well to avoid computing them again multiple times later.

In [12]:

```
filtered = data.toTimeSeries().detrend('nonlinear').normalize()
```

Perform the regression

In [13]:

```
results = model.fit(X.T, filtered)
```

Look at the first record to confirm that the operation works

In [14]:

```
results.stats.first()
```

Out[14]:

```
((0, 0, 0), 0.002423701256132671)
```

Now run on the entire data set, and put the results into a local array

In [15]:

```
r2 = results.stats.pack()
```

Use a maximum intensity projection to look at R2

In [16]:

```
from numpy import amax
image(amax(r2, axis=2).T, clim=(0, 0.4), size=20)
```

Out[16]:

```
<matplotlib.image.AxesImage at 0x3d4cb90>
```

# Tuning¶

When we did the regression, we estimated 12 regression weights per pixel. To reduce these 12 numbers to a single number, we use the fact that those 12 weights correspond to 12 different turning angles, which we stored with our parameters.

In [17]:

```
angles = params['theta']
```

Now construct and fit a tuning model

In [18]:

```
from thunder import TuningModel
tuning = TuningModel.load(angles, 'gaussian')
estimates = tuning.fit(results.coeffs.between(1,12))
```

pack the estimated tuning into a local array

In [19]:

```
tuningvals = estimates.select('center').pack()
```

We can look at a single plane with these tuning values, but it's not particularly useful because voxels will appear colored regardless of the reliability of their responses

In [20]:

```
image(tuningvals[:,:,17].T, cmap='gist_rainbow', size=20)
```

Out[20]:

```
<matplotlib.image.AxesImage at 0x3e05bd0>
```

And let's compute the mean of the raw data over time as a background image

In [21]:

```
base = data.seriesMean().pack()
```

We can use Thunder's colorization to merge the tuning values, background, and r2 (as a mask) into a composite image

In [23]:

```
from thunder import Colorize
clr = Colorize(cmap='gist_rainbow')
mapplane = clr.transform(tuningvals[:,:,17], mask=r2[:,:,17], background=base[:,:,17], mixing=0.75)
```

Look at the map for a single plane

In [24]:

```
image(mapplane.swapaxes(0,1), size=20)
```

Out[24]:

```
<matplotlib.image.AxesImage at 0x6cb8b650>
```

Now do the colorization on a volume

In [25]:

```
mapvolume = clr.transform(tuningvals, mask=r2, background=base, mixing=0.75)
```

And compute the maximum projection

In [26]:

```
image(amax(mapvolume, axis=2).swapaxes(0,1), size=20)
```

Out[26]:

```
<matplotlib.image.AxesImage at 0x6cb94c90>
```
